# Comparative analysis of clinical and imaging data of first-attack neuromyelitis optica spectrum disorders with and without connective tissue disease

**DOI:** 10.3389/fneur.2022.969762

**Published:** 2022-08-25

**Authors:** Yaobing Yao, Xuan Yang, Yongyan Zhou, Haojie Xie, Ranran Duan, Lijun Jing, Yanfei Li, Wenjuan Guan, Junfang Teng, Yanjie Jia

**Affiliations:** ^1^Department of Neurology, The First Affiliated Hospital of Zhengzhou University, Zhengzhou, China; ^2^Department of Rheumatology, The First Affiliated Hospital of Zhengzhou University, Zhengzhou, China

**Keywords:** neuromyelitis optica spectrum disorder, connective tissue disease, relapse, homocysteine, risk factors

## Abstract

**Background:**

The coexistence of neuromyelitis optica spectrum disorder (NMOSD) and connective tissue disease (CTD) is well recognized. The purpose of this study was to investigate and compare the characteristics of first attack NMOSD with and without CTD.

**Methods:**

A total of 113 Patients with NMOSD were included and were divided into two groups based on the presence of co-occurring CTD. Their demographic, clinical, laboratory, and image characteristics were obtained through inpatient medical records and follow-ups. Kaplan–Meier survival analysis was used to analyze the effect of CTD in NMOSD patients at the time of first recurrence. The risk factors that could predict complications of NMOSD with CTD was analyzed by binary logistic regression. The ability of homocysteine (Hcy) to predict the coexistence of NMOSD and CTD was analyzed and evaluated by the receiver operating characteristic curve.

**Results:**

The demographic data, clinical features, cerebrospinal fluid analysis, and MRI findings, except relapse events (including relapse rate, number of recurrences, and time of first recurrence), were similar between the two groups. The serum lymphocyte-to-monocyte ratio and albumin levels were lower (*P* < 0.05), while serum erythrocyte sedimentation rate and Hcy levels were higher in patients with NMOSD with CTD than in those without CTD (*P* < 0.001). Kaplan–Meier survival analysis showed that the time of first recurrence in NMOSD patients complicated with CTD was earlier than that of without CTD (log rank test *P* = 0.035). Logistic regression revealed that serum Hcy levels (OR 1.296, 95% CI, 1.050–1.601, *P* = 0.016) were independently associated with the occurrence of NMOSD with CTD. The receiver operating characteristic curve area was 0.738 (95% CI, 0.616–0.859; *P* < 0.001) for Hcy levels. Considering the Hcy concentration of 14.07 μmol/L as the cutoff value, the sensitivity and specificity of predicting the coexistence of first-attack NMOSD and CTD were 56 and 89.8%, respectively.

**Conclusions:**

When the first-attack NMOSD patients are complicated with CTD, they have a higher recurrence rate, more recurrences, earlier first recurrence, higher serum Hcy levels, and enhanced systemic inflammatory reactions. Furthermore, Hcy levels may help to screen for CTD in patients with first-attack NMOSD.

## Introduction

Aquaporin-4 (AQP4) was found in 2004 as a water channel that is expressed at high concentration on the foot processes of perivascular astrocytes (1, 2). This landmark discovery marked a new era for neuromyelitis optica. With better understanding of the pathophysiology of neuromyelitis optica, more accurate diagnostic criteria, new nomenclature (“neuromyelitis optica spectrum disorder”, NMOSD), and new treatment strategies have been developed (3). The central clinical symptoms of NMOSD include the loss of vision from optic neuritis, spinal cord syndrome from the myelitis, intractable nausea, vomiting, and hiccups from the posterior polar zone syndrome (4). Other clinical syndromes caused by damage including hypothalamus and brain lesions are not common (5). Many patients with NMOSD face adverse consequences of recurrent disease and increased disability after their first onset.

Connective tissue disease (CTD) is a large class of rheumatic diseases related to autoimmune dysfunction that can cause damage to multiple organs and systems (6). Although demyelination of the central nervous system can be caused by CTD alone, the coexistence of CTD and NMOSD has been clearly recognized by neurologists and rheumatologists (7). The combination of these two diseases is likely to affect the treatment and prognosis of the patients (7). Unlike the white populations in Europe and North America, the incidence of NMOSD is significantly higher than that of multiple sclerosis in East Asia (8, 9). In the current series of clinical studies on NMOSD with CTD, only a few single-center small-scale studies have been conducted mainly in East Asia (10–15). It is known from these studies that patients with both NMOSD and CTD may require unique diagnosis and treatment. It is very important to identify the coexistence of the two diseases earlier and actively adjust the treatment plan to reduce the recurrence of the disease. Therefore, further research is required. In this single-center retrospective cohort study, we aimed to investigate and compare the demographic, clinical, laboratory, and image characteristics of patients with first-attack NMOSD with and without CTD.

## Methods

### Patients

**Figure 1:**
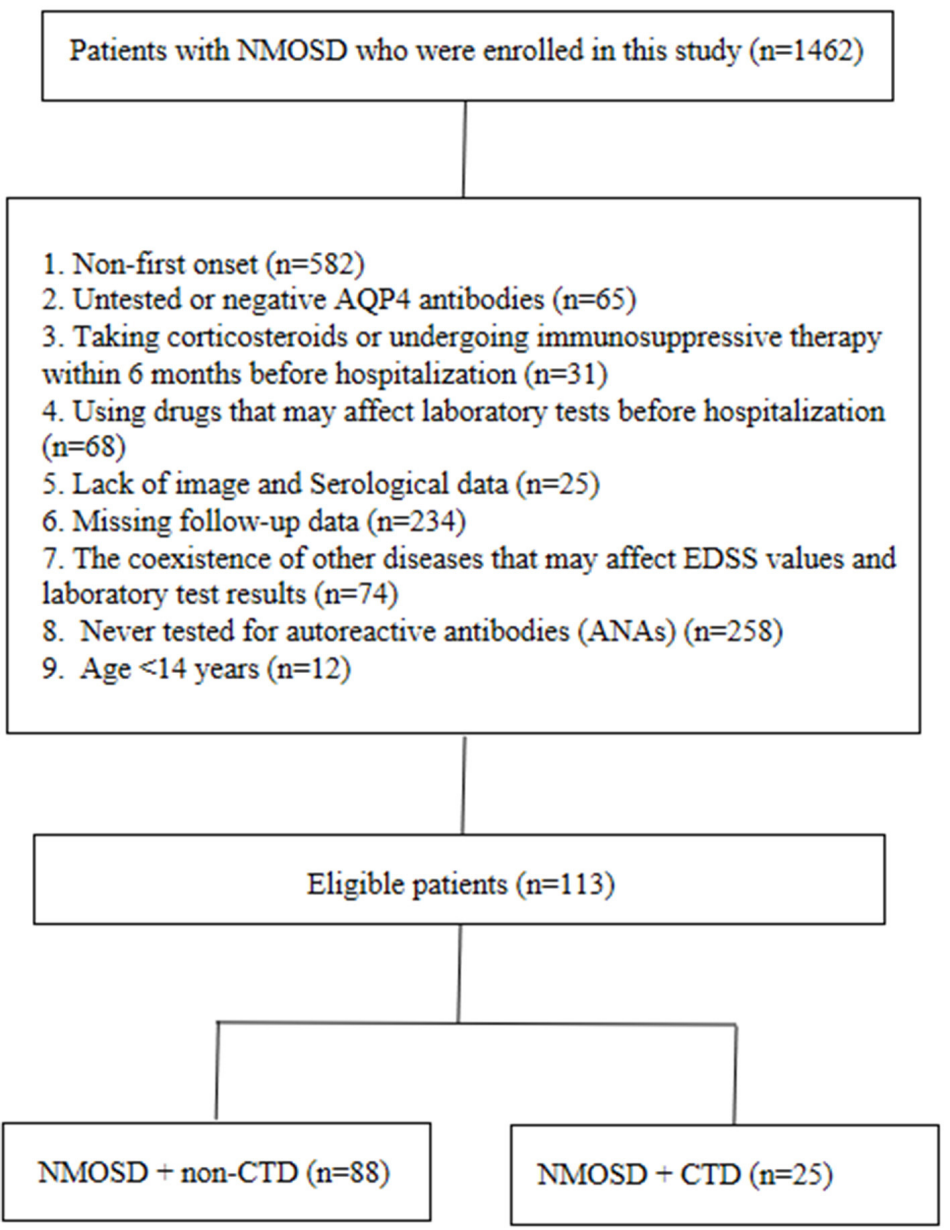
Patient flowchart.

This retrospective cohort study included patients with NMOSD who were treated at the Department of Neurology of the first affiliated Hospital of Zhengzhou University from January 2013 to December 2021. NMOSD was diagnosed based on NMOSD's 2015 international consensus diagnostic criteria (4). A NMOSD relapse event was defined as new-onset or recurrent neurological symptoms that lasted for at least 24 h and caused an EDSS increase of at least 0.5 points from the lowest score. Relapse events occurring within 28 days were regarded as part of a single relapse (16). CTD was diagnosed by rheumatologists according to the published standards and classification guidelines [e.g., systemic lupus erythematosus (SLE), Sjogren's syndrome (SS), Behcet's disease, and rheumatoid arthritis] (17–20). We excluded patients with NMOSD according to the following criteria: (1) non-first onset; (2) untested or negative AQP4 antibodies (AQP4-IgG); (3) corticosteroid or immunosuppressive therapy within 6 months before hospitalization; (4) use of drugs that may affect laboratory tests, such as B vitamins, lipid-lowering medicines, and hepatorenal protectants before hospitalization; (5) lack of image and serological data; (6) missing follow-up data; (7) coexistence of other diseases that may affect the Extended Disability Status Scale (EDSS) values and laboratory test results; (8) patients never tested for antinuclear antibodies (ANA); and (9) age <14 years. The detailed selection process is shown in Figure 1. The study was approved by the Ethics Committee of the First Affiliated Hospital of Zhengzhou University (2019-KY-018) and conducted in accordance with the Declaration of Helsinki. All the study participants signed the informed consent forms.

### Data collection

We collected the following data on patients with NMOSD: demographic characteristics (age and sex), past medical history (hypertension, diabetes mellitus, and coronary heart disease), EDSS (at admission, discharge, and follow-up), relapse, clinical manifestations (vision loss, physical mobility disorder, paresthesia, nausea/vomiting, dizziness, pain, fever, abnormal bowel, and urinary function), blood test results [glucose, blood routine, hepatorenal function, blood clotting function, blood lipid, thyroid hormone test, erythrocyte sedimentation rate (ESR), C-reactive protein, and homocysteine (Hcy)], cerebrospinal fluid (CSF) test results (cell count, lymphocyte proportion, protein levels, albumin quotient, and immunoglobulin quotient), and characteristics of magnetic resonance imaging (MRI). Blood and CSF samples were collected before treatment. Data were obtained through inpatient medical records and quarterly follow-ups after discharge. AQP4-IgG was measured by cell-based assay in the serum or CSF (detailed testing methods and criteria for judging the results can be found in Supplementary material 1).

### Statistical analysis

We used SPSS software (version 25.0; IBM, Armonk, NY, USA) to analyze the study data. The normal test was used for the data, and the normal distribution data are expressed as mean ± standard deviation. Independent sample *t*-tests were used to compare the two groups of data. The data with non-normal distribution are represented as medians (P_25_, P_75_) and compared using the Mann-Whitney U test. Classification data are presented as number of cases (percentage) and compared using the chi-square test or Fisher's exact test. A Kaplan–Meier analysis and log-rank test were used to analyze the effect of CTD in NMOSD patients at the time of first recurrence. We used regression analyses to determine factors that could distinguish between two groups. The ability of Hcy to predict the coexistence of NMOSD and CTD was analyzed and evaluated by the receiver operating characteristic (ROC) curve, which was generated using SPSS software. The best cutoff point, with the highest sum of sensitivity and specificity, was also detected. The area under the curve was calculated based on ROC curve analysis. All statistical significances were set at *P* < 0.05 and statistical analyses were 2-tailed.

## Results

### Demographic and clinical characteristics

A total of 113 patients with NMOSD who met the criteria were enrolled in this study; 88 did not have CTD and 25 had CTD (Figure 1). Table 1 summarizes the demographic and clinical characteristics of patients with NMOSD with CTD (16 patients with SS, 7 patients with SLE, 1 patient with RA, and 1 patient with BD). Among these patients with CTD, 8 were diagnosed before the onset of NMOSD (ranging from 4 months to 12 years), 9 were diagnosed when NMOSD occurred, and the other 8 were diagnosed after the onset of NMOSD (ranging from 2 months to 3 years).

**Table 1:** Demographic and clinical characteristics in patients of first-attack NMOSD with CTD.

**Variables**	**SS**	**SLE**	**BD**	**RA**
	**(*N* = 16)**	**(*N* = 7)**	**(*N* = 1)**	**(*N* = 1)**
Sex (female)	13 (81.25%)	7 (100%)	0 (0%)	1 (100%)
Age (years)	41.88 ± 12.06	38.86 ± 13.43	30.0 ± 0	48.0 ± 0
Duration of follow-up (months)	41.0 (29.0, 78.75)	41.0 (27.0, 81.00)	45.0 (45.0, 45.0)	72.0 (72.0, 72.0)
Relapse	12 (75.0%)	5 (71.43%)	1 (100.0%)	1 (100.0%)
Admission EDSS	5 (3, 6)	8 (4, 9)	3 (3, 3)	3 (3, 3)
Discharge EDSS	3 (3, 4.75)	8 (3, 8)	3 (3, 3)	2 (2, 2)
Follow-up EDSS	3.5 (2, 5)	3 (3, 7)	3 (3, 3)	3 (3, 3)
Myelitis	11 (68.75%)	4 (57.14%)	1 (100.0%)	1 (100.0%)
Optic neuritis	3 (18.75%)	2 (28.57%)	0 (0.0%)	0 (0.0%)
Area postrema syndrome	0 (0.0%)	2 (28.57%)	0 (0.0%)	0 (0.0%)
**Diagnosis sequence of CTD compared with NMOSD**				
Before	5 (31.25%)	2 (28.57%)	0 (0.0%)	1 (100.0%)
Meanwhile	6 (37.50%)	2 (28.57%)	1 (100.0%)	0 (0.0%)
After	5 (31.25%)	3 (42.86%)	0 (0.0%)	0 (0.0%)

Values are presented as the mean ± SD, numbers, or median (interquartile range).

NMOSD, neuromyelitis optica spectrum disorders; CTD, connective tissue disorders; SS, Sjogren's syndrome; SLE, systemic lupus erythematosus; BD, Behcet's disease; RA, rheumatoid arthritis; EDSS, kurtzke expanded disability status scale.

There was no difference in demographic and clinical features, except relapse events, between the two groups. The serum lymphocyte-to-monocyte ratio (LMR) and albumin levels were lower (*P* < 0.05), while serum ESR and Hcy levels were significantly higher in patients with NMOSD with CTD than in those without CTD (*P* < 0.001). There were no other differences in CSF analysis or MRI findings between the two groups.

Table 2 summarizes and compares the demographic and clinical characteristics of the two groups. The relapse rate was significantly higher in patients with NMOSD with CTD than in those without CTD (*P* = 0.016). There were no other differences in demographics and clinical features, except relapse events (including relapse rate, number of recurrences, and time of first recurrence), between the two groups (*P* > 0.05). Table 3 summarizes and compares the results of CSF analysis between the two groups. No significant differences were noted in white blood cell count (*P* = 0.764), lymphocyte count (*P* = 0.560), protein quantity (*P* = 0.402), albumin quotient (*P* = 0.408), immunoglobulin G quotient (*P* = 0.559), or oligoclonal band positivity (*P* = 0.193) between the two groups. Table 4 summarizes and compares the blood test results of the two groups. Our findings indicated that LMR (median: 4.00 vs. 3.06, *P* = 0.007) and serum albumin levels (average: 41.37 vs. 39.36 g/L, *P* = 0.049) were lower in patients with NMOSD with CTD than in those without CTD. In contrast, ESR (average: 10.00 vs. 18.00 mm/h, *P* < 0.001) and Hcy levels (median: 10.43 vs. 15.30 μmol/L, *P* < 0.001) were significantly higher in patients with NMOSD with CTD than in those without CTD. Table 5 summarizes and compares the MRI characteristics of the two groups. There were no significant differences in MRI findings between the two groups (*P* > 0.05). In particular, no differences in length of spinal cord lesions were found between the two groups, as previously reported (*P* = 0.882) (13).

**Table 2:** Demographic and clinical characteristics in patients of first-attack NMOSD with and without CTD.

**Variables**	**NMOSD + non-CTD (*N* = 88)**	**NMOSD + CTD (*N* = 25)**	***P*-value**
Sex (female)	56/88 (63.64%)	21/25 (84.0%)	0.054
Age (years)	45.17 (84.0%)	40.80 (84.0%)	0.177
Admission EDSS	5 (3, 6)	5(3, 7.8)	0.337
Discharge EDSS	4 (2, 5.9)	3 (3, 7)	0.504
Follow-up EDSS	3 (2, 4)	3 (2.5, 5)	0.266
Duration of follow-up (months)	45.50 (31.25, 58.0)	42.0 (29.0, 80.0)	0.246
Relapse	43 (48.86%)	19 (76.0%)	0.016
Number of recurrence	3(2, 4)	6 (5, 7)	<0.001
Time of first recurrence	15.1 (6.3, 21.6)	6.6 (3.9, 9.8)	0.035
Hypertension	6 (6.82%)	2 (8.0%)	1.000
Diabetes	5 (5.68%)	0 (0.0%)	0.504
Coronary heart disease	5 (5.68%)	0 (0.0%)	0.504
Myelitis	46 (52.27%)	17 (68.0%)	0.162
Optic neuritis	32 (36.36%)	5 (20.0%)	0.124
Area postrema syndrome	10 (11.36%)	2 (8.0%)	0.909
ADEM	0 (0.0%)	0 (0.0%)	—
Brainstem syndrome	19 (21.59%)	2 (8.0%)	0.211
Myelitis and optic neuritis	14 (15.90%)	1 (4.0%)	0.224
Myelitis and brainstem syndrome	7 (7.95%)	2 (8.0%)	1.000
Optic neuritis and brainstem syndrome	6 (6.82%)	0 (0.0%)	0.403
Myelitis and area postrema syndrome	7 (7.95%)	1 (4.0%)	0.811
Optic neuritis and area postrema syndrome	3 (3.41%)	0 (0.0%)	1.000
Vision loss	28 (31.82%)	4 (16.0%)	0.121
Physical mobility disorder	12 (13.64%)	8 (32.0%)	0.068
Paresthesia	36 (40.91%)	15 (60.0%)	0.091
Nausea/vomiting	5 (5.68%)	2 (8.0%)	1.000
Dizziness	7 (7.95%)	5 (20.0%)	0.175
Pain	13 (14.77%)	1 (4.0%)	0.272
Abnormal bowel and urinary function	4 (4.55%)	3 (12.0%)	0.371
Disturbance of consciousness	1 (1.14%)	0 (0.0%)	1.000
Fever	5 (5.68%)	1 (4.0%)	1.000

Values are presented as the mean i SD, numbers, or median (interquartile range).

Statistical significance with two-sided p < 0.05.

AQP4, aquaporin-4; NMOSD, neuromyelitis optica spectrum disorder; CTD, connective tissue disease; EDSS, extended disability status scale; ADEM, acute disseminated encephalomyelitis.

**Table 3:** Cerebrospinal fluid (CSF) analysis in patients of AQP4-positive NMOSD with and without CTD.

**Variables**	**NMOSD + non-CTD (*N* = 61)**	**NMOSD + CTD (*N* = 15)**	***P*-value**
CSF white blood cell count (Normal value: 0nt × 106/L)	6 (2, 14)	4 (2, 12)	0.764
CSF lymphocyte proportion (Normal value: 60al va	77 (68, 84)	80 (61, 85)	0.560
CSF protein (Normal value: 15060al value	367.50 (262.25, 519.75)	389.70 (220.70, 470.0)	0.402
QALB	4.98 (3.71, 7.57)	5.68 (4.15, 10.43)	0.408
QIgG	3.66 (2.56, 5.59)	3.42 (2.01, 4.86)	0.559
Positive oligoclonal band	6/61 (9.84%)	4/15 (26.67%)	0.193

Values are presented as numbers or the median (interquartile range).

Statistical significance with two-sided p < 0.05.

AQP4, aquaporin-4; NMOSD, neuromyelitis optica spectrum disorder; CTD, connective tissue disease; QALB, albumin quotient; QIgG, immunoglobulin G quotient.

**Table 4:** Blood tests results in patients of first-attack NMOSD with and without CTD.

**Variables**	**NMOSD + non-CTD (*N* = 88)**	**NMOSD + CTD (*N* = 25)**	***P*-value**
NLR	2.58 (1.67, 3.35)	2.61 (1.75, 7.89)	0.271
LMR	4.0 (2.84, 5.0)	3.06 (1.98, 3.99)	0.007
PLR	131.23 (97.90, 201.61)	180.49 (96.58, 273.70)	0.061
Glucose (mmol/L)	4.60 (4.25, 5.24)	4.64 (4.23, 6.33)	0.614
Albumin (g/L)	41.37 ± 4.64	39.36 ± 3.80	0.049
Total bilirubin (μ mmol/L)	9.50 (6.16, 13.08)	8.10 (6.30, 11.50)	0.382
Uric acid (μ mmol/L)	208.50 (173.50, 255.0)	179.0 (165.0, 242.50)	0.219
Creatinine (μ mmol/L)	54.09 ± 11.99	53.37 ± 14.98	0.801
GFR (ml/min)	112.42 ± 13.61	115.69 ± 14.99	0.302
Total cholesterol (mmol/L)	4.38 (3.69, 4.85)	4.21 (3.79, 4.45)	0.287
Triglycerides (mmol/L)	1.20 (0.84, 1.57)	1.11 (0.77, 1.36)	0.296
High-density lipoprotein cholesterol (mmol/L)	1.20 (1.06, 1.41)	1.23 (1.04, 1.50)	0.827
Low-density lipoprotein cholesterol (mmol/L)	2.66 (2.17, 3.18)	2.74 (2.35, 3.18)	0.920
ESR (mm/h)	10.0 (7.40, 13.0)	18.0 (12.0, 28.0)	<0.001
CRP (mg/L)	1.52 (0.81, 2.85)	2.53 (1.07, 4.87)	0.111
FT3 (pmol/L)	4.54 (3.99, 5.06)	4.46 (4.0, 4.84)	0.406
FT4 (pmol/L)	11.34 (10.26, 12.36)	11.59 (9.62, 12.83)	0.676
HCY (μ mmol/L)	10.43 (8.63, 12.19)	15.30 (11.12, 21.24)	<0.001
Prothrombin time (s)	10.35 (9.80, 10.80)	10.40 (9.60, 10.75)	0.745
Prothrombin temporal activity (%)	108.0 (94.20, 117.98)	105.0 (98.45, 118.50)	0.983
INR	0.94 (0.89, 1.0)	0.94 (0.86, 1.0)	0.618
APTT (s)	29.60 (26.80, 32.50)	29.30 (27.10, 33.50)	0.787
Fibrinogen (g/L)	2.86 (2.34, 3.40)	2.93 (2.59, 3.76)	0.367
Thrombin time (s)	14.30 (13.30, 15.50)	14.50 (13.85, 15.30)	0.582
D-dimer (mg/L)	0.12 (0.06, 0.21)	0.12 (0.65, 0.31)	0.426
FDP (μ g/mL)	1.24 (0.95, 2.05)	1.17 (0.71, 2.92)	0.822

Values are presented as mean ± SD, numbers, or the median (interquartile range).

Statistical significance with two-sided p < 0.05.

AQP4, aquaporin-4; NMOSD, neuromyelitis optica spectrum disorder; CTD, connective tissue disorders; NLR, neutrophil-lymphocyte ratio; LMR, lymphocyte-to-monocyte ratio; PLR, platelet-lymphocyte ratio; GFR, glomerular filtration rate; ESR, erythrocyte sedimentation rate; CRP, C-reactive protein; Hcy, homocysteine; INR, international normalized ratio; APTT, activated partial prothrombin time; FDP, fibrinogen degradation products.

**Table 5:** MRI findings in patients of first-attack NMOSD with and without CTD.

**Variables**	**NMOSD + non-CTD (*N* = 88)**	**NMOSD + CTD (*N* = 25)**	***P*-value**
**Cranial MRI**			
Cortical gray matter/juxtacortical white matter	11 (12.50%)	4 (16.0%)	0.904
Midbrain	1 (1.14%)	1 (4.0%)	0.395
Pons	7 (7.95%)	5 (20.0%)	0.175
Thalamus	7 (7.95%)	2 (8.0%)	1.000
Cerebellar hemisphere	6 (6.82%)	0 (0.0%)	0.403
Basal ganglia	2 (2.27%)	2 (8.0%)	0.212
Callosal	0	1 (4.0%)	0.221
Lateral ventricle	5 (5.68%)	4 (16.0%)	0.207
Medulla oblongata	21 (23.86%)	5 (20.0%)	0.685
**Spinal MRI**			
Cervical cord	48 (54.55%)	13 (52.0%)	0.822
Thoracic cord	50 (56.82%)	13 (52.0%)	0.669
Spinal cord involvement, segment number	5 (3, 9)	6 (3, 8)	0.882
**Optic nerve MRI**			
Optic nerve	6 (6.82%)	5 (20.0%)	0.114
Optic chiasm	4 (4.55%)	3 (12.0%)	0.371
Optic tract	4 (4.55%)	2 (8.0%)	0.862
Lesion adjacent to the body of an LV and inferior temporal lobe lesion	5 (5.68%)	2 (8.0%)	1.000
U-fiber lesions	2 (2.27%)	1 (4.0%)	0.531
Dawson's fingers	1 (1.14%)	1 (4.0%)	0.395
ALL^*^	1 (1.14%)	1 (4.0%)	0.395

Values are presented as numbers or the median (interquartile range).

Statistical significance with two-sided p < 0.05.

* ALL refers to the combination of a lesion adjacent to the body of an LV and an inferior temporal lobe lesion, U-fiber lesions, and Dawson's fingers.

AQP4, aquaporin-4; NMOSD, neuromyelitis optica spectrum disorder; CTD, connective tissue disease; MRI, magnetic resonance imaging; LV, Lateral ventricle.

### Kaplan–Meier analysis for the time of first recurrence in patients with recurrent NMOSD

**Figure 2:**
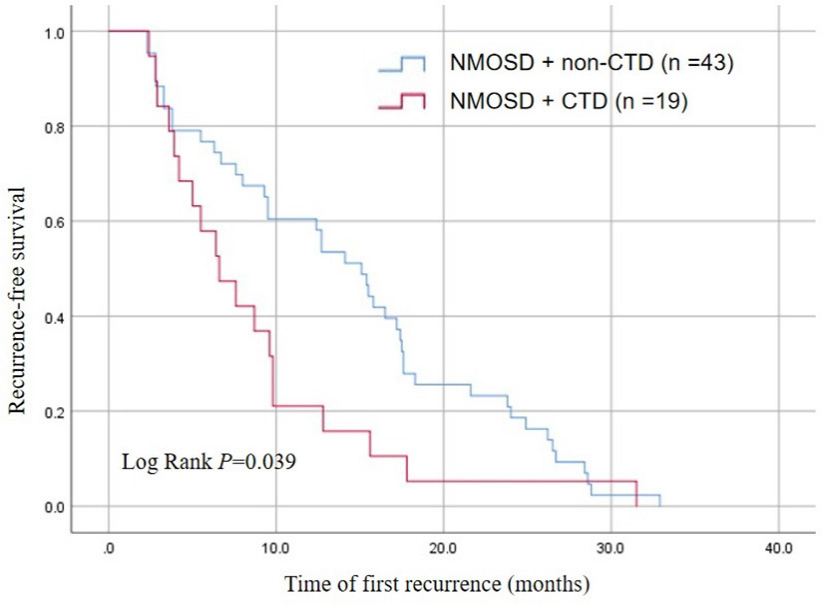
Kaplan-Meier analysis demonstrating the cumulative proportion of recurrent patients without relapse in the NMOSD + non-CTD group and the NMOSD + CTD group.

Kaplan–Meier survival analysis showed that the time of first recurrence in NMOSD patients complicated with CTD was significantly earlier than in those without CTD (log rank test *P* = 0.035). The median relapse interval in the NMOSD + CTD group was 6.6 months, and the relapse rate within 12 months was 60.0%. In contrast, the median relapse interval in the NMOSD + non-CTD group was 15.1 months, and the relapse rate within 12 months was only 19.32% (Figure 2).

### Prediction of NMOSD complicated with CTD

Univariable logistic regression analyses for risk factors that could predict complications of NMOSD with CTD showed that LMR (OR 0.668, 95% CI 0.491–0.911, *P* = 0.011), ESR (OR 1.035, 95% CI 1.007–1.035, *P* = 0.053), and Hcy levels (OR 1.157; 95% CI 1.065–1.257; *P* = 0.001) were associated with the coexistence of NMOSD and CTD (Table 6).

**Table 6:** Univariable logistic regression analysis of factors differentiating patients of first-attack NMOSD with and without CTD.

**Variables**	***B***	**SE**	**Wald**	***P*-value**	**EXP(B)**	**EXP(B) 95% CI**	
						**Lower**	**Upper**
LMR	−0.403	0.158	6.508	0.011	0.668	0.491	0.911
Albumin (g/L)	−1.00	0.052	3.734	0.053	0.904	0.817	1.001
ESR (mm/h)	0.034	0.014	5.896	0.015	1.035	1.007	1.064
Hcy (μmol/L)	0.146	0.042	11.867	0.001	1.157	1.065	1.257

NMOSD, neuromyelitis optica spectrum disorder; CTD, connective tissue disorders; SE, standard error; CI, confidence interval; LMR, lymphocyte-to-monocyte ratio; ESR, erythrocyte sedimentation rate; Hcy, homocysteine.

After univariable logistic regression analysis, the variables with a significance of *P* < 0.05, as well as age, sex, and clinical variables that may be predictors of CTD in patients with NMOSD were entered into the multivariable model. The basic model of multivariable logistic regression analysis showed that serum Hcy levels (OR 1.129, 95% CI, 1.036–1.230, *P* = 0.006) were significantly associated with the coexistence of NMOSD and CTD (Table 7). In the adjusted model^c^ (with the lowest Akaike Information Criteria), serum Hcy levels (OR 1.296, 95% CI, 1.050–1.601, *P* = 0.016) remained significantly associated with the coexistence of NMOSD and CTD (Table 7).

**Table 7:** Multivariable logistic regression analysis of factors differentiating patients of first-attack NMOSD with and without CTD.

	**Basic model** ^**a**^				**Adjusted model** ^**b**^				**Adjusted model** ^**c**^			
**AIC**	**107.613**				**107.954**				**77.551**			
**Variables**	***P*** **-value**	**EXP(B)**	**EXP(B) 95% CI**		***P*** **-value**	**EXP(B)**	**EXP(B) 95% CI**		***P*** **-value**	**EXP(B)**	**EXP(B) 95% CI**	
			**Lower**	**Upper**			**Lower**	**Upper**			**Lower**	**Upper**
Age					0.343	0.981	0.943	1.021	0.850	1.006	0.947	1.068
Sex					0.138	0.378	0.105	1.367	0.215	0.270	0.034	2.143
LMR	0.144	0.785	0.567	1.086	0.205	0.809	0.582	1.123	0.341	0.804	0.514	1.259
Albumin (g/L)									0.158	0.858	0.694	1.061
Uric acid (μmol/L)									0.468	1.005	0.992	1.018
ESR (mm/h)	0.186	1.021	0.990	1.053	0.218	1.019	0.989	1.051	0.736	1.008	0.964	1.053
CRP (mg/L)									0.871	0.996	0.945	1.049
FT4 (pmol/L)									0.621	1.083	0.790	1.484
Hcy (μmol/L)	0.006	1.129	1.036	1.230	0.006	1.132	1.036	1.236	0.016	1.296	1.050	1.601

NMOSD, neuromyelitis optica spectrum disorder; CTD, connective tissue disease; AIC, Akaike Information Criteria; CI, confidence interval; LMR, lymphocyte-to-monocyte ratio; ESR, erythrocyte sedimentation rate; CRP, C-reactive protein; Hcy, homocysteine.

Basic Model^a^: Variables with P < 0.05 in the univariable logistic regression analysis were included in the multivariable model.

Adjusted Model^b^: Age, sex and variables with P < 0.05 in the univariable logistic regression analysis were included in the multivariable model.

Adjusted Model^c^: Age, sex and variables with P < 0.05 in the univariable logistic regression analysis or variables clinically may be potential predictors of CTD in patients with NMOSD, after investigating multicollinearity between the independent variables (Supplementary material 2), were included in the adjusted model.

### ROC curve for Hcy at admission to predict the coexistence of NMOSD and CTD

**Figure 3:**
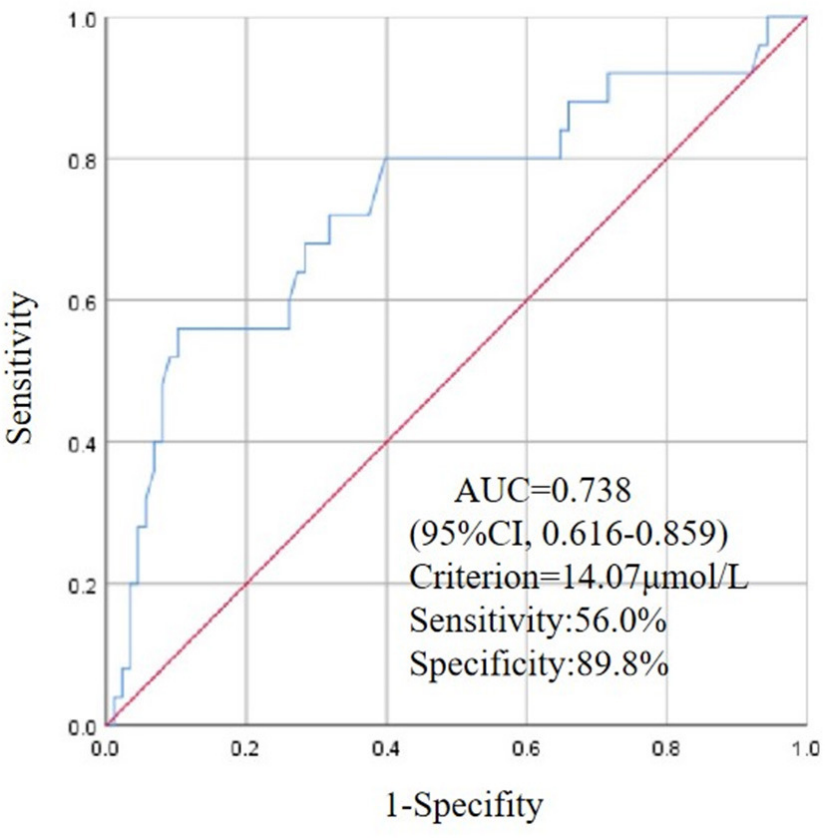
ROC curve for values of HCY to predict CTD in patients of first-attack NMOSD.

We used the ROC curve to analyze and evaluate the predictive value of Hcy in patients with NMOSD accompanied by a CTD. The area under the ROC curve of Hcy levels was 0.738 (95% CI, 0.616–0.859; *P* < 0.001). Considering the homocysteine concentration of 14.07 μmol/L as the cutoff value, the sensitivity and specificity of predicting the coexistence of first-attack NMOSD and CTD were 56 and 89.8%, respectively (Figure 3).

## Discussion

In this study, we found that the demographic data and clinical features, except for relapse events (including relapse rate, number of recurrences, and time of first recurrence), were similar between patients with first-onset NMOSD with CTD and those without CTD. Furthermore, Kaplan-Meier survival analysis showed that the time of first recurrence in NMOSD patients complicated with CTD was significantly earlier than in patients without CTD. Additionally, MRI features and CSF analysis did not show significant differences between the two groups. However, several blood test results, including those for LMR, albumin, ESR, and Hcy, differed significantly between the groups. Logistic regression analysis indicated that Hcy levels might be independent risk factors for concurrent CTD in patients with first-attack NMOSD. The optimal cutoff value of Hcy for predicting NMOSD complicated by CTD was 14.07 μmol/L.

NMOSD is a common autoimmune disease of the central nervous system, mainly affecting the optic nerve and spinal cord (3). CTD is a large class of autoimmune diseases that can cause damage to multiple organs and systems, including the nervous system, such as SLE, RA, SS, and BD (6, 7). NMOSD and CTD have many repetitive clinical features that can lead to severe and irreversible disabilities (7). Large-scale data analysis showed that the incidence of NMOSD in China was 0.278/100,000 person-years (21). SS (6.5%) and SLE (2.2%) were the most common autoimmune diseases associated with NMOSD (21). In the context of NMOSD core clinical syndrome, the presence of SS and SLE reinforces and supports a diagnosis of NMOSD (4). In addition, NMOSD with CTD is an independent risk factor for a high recurrence rate within a year (16). Our analysis revealed that the recurrence rate within 12 months in the NMOSD+CTD group was 60.0%, while it was only 19.32% in the NMOSD+ non-CTD group (Figure 2). Furthermore, We found that the time of first recurrence in NMOSD patients complicated with CTD was significantly earlier than in patients without CTD (Figure 2). What is of great significance was that previous studies about the coexistence of CTD and NMOSD have not mentioned this aspect.

Physicians treating patients with co-occurring NMOSD with CTD should adopt customized treatment strategies to reduce disease recurrence (22–27). In short, clinicians need to diagnose NMOSD and CTD as two separate entities in patients with concomitant diseases. Antibody spectrum detection [e.g., ANA, anti-SSA/Ro antibodies (anti-SSA), anti-SSB/La antibodies (anti-SSB), anti-double-stranded DNA antibodies, and rheumatoid factor] can play important roles in the diagnosis of CTD, especially SS and SLE (6). The anti-SSA/Ro antibody could be associated with disease activity and severe disability in NMOSD (28). However, because many neurologists have insufficient information on NMOSD with CTD, only a very small number of patients with NMOSD have been tested for ANA or the other antibodies mentioned above (Figure 1). In patients with NMOSD without autoimmune diseases, these autoantibodies were also discovered, but lower levels were observed than those in patients with concomitant autoimmune processes (15, 29). This phenomenon was also observed in this study (not specified). In many previously published articles, rheumatologists have recommended that serum AQP4 and myelin oligodendrocyte glycoprotein antibodies should be considered for all patients with CTD with myelitis, bilateral optic neuritis, and large fusion brain damage (30–34). Similarly, neurologists should consider testing for these autoantibodies in patients with NMOSD. The consensus of experts suggests that neurologists should screen for these autoantibodies in patients with NMOSD only if their clinical symptoms suggest that they may be complicated with CTD (7). Therefore, neurologists need to include a detailed history of CTD symptoms in their preliminary assessments. This does not apply to many patients with asymptomatic CTD having overlapping symptoms of NMOSD. Fortunately, the results of this study may help solve this problem.

Hcy is a commonly used serological index in clinics, and its application is both economical and extensive. Elevated levels of Hcy can damage the blood-brain barrier and promote myelin degeneration (35, 36). Measuring Hcy levels is becoming more and more important in patients with inflammatory demyelinating neurological disorders. Two meta-analysis showed multiple sclerosis patients tend to have elevated Hcy blood levels compared to healthy controls (37, 38). Clinical studies have shown that patients with multiple sclerosis can benefit from a reduction in Hcy (39). Our research group has also reported two important findings regarding the role of Hcy in NMOSD (21). The first finding was that the Hcy level might be an independent risk factor for AQP4-IgG-positive highly active NMOSD. The second finding was that the Hcy level could predict relapse and prognosis in patients with first-attack NMOSD (40). In addition, another research team found that plasma Hcy levels were associated with EDSS in NMOSD (41). Although the mechanism of central nervous system damage caused by higher Hcy levels in patients with NMOSD-CTD is unclear, lowering the level of Hcy is likely to be beneficial to patients. In this study, patients with first-attack NMOSD with CTD had higher Hcy levels than those without CTD, and logistic regression revealed that serum Hcy level was an independent predictor of the occurrence of NMOSD with CTD. The area under the ROC curve of Hcy levels was 0.738. Considering the Hcy concentration of 14.07 μmol/L as the cutoff value, the sensitivity and specificity of predicting the coexistence of first-attack NMOSD and CTD were 56 and 89.8%, respectively. Therefore, this study suggests that ANA and other antibodies should be detected when the serum Hcy level in patients with first-attack NMOSD is higher than 14.07 μmol/L. This discovery will help screen patients with CTD with overlapping symptoms of NMOSD and asymptomatic patients.

In this study, 25 patients with CTD were included, of which 21 (84%) were women. In 8 (32%) patients, NMOSD was followed by CTD onset; in 9 (36%) patients, the presentation was simultaneous; and in 8 (32%) patients, NMOSD preceded CTD onset. This finding is similar to that reported previously (29). This supports the notion that NMOSD and CTD are two independent disease entities that coexist easily in clinics. Therefore, it was considered that in patients with NMOSD and CTD, central nervous system damage is secondary to astrocytic lesions caused by AQP4-IgG rather than vasculitis in SS or SLE (42). LMR, ESR, and CRP are markers of systemic inflammation and immune response in CTD (43, 44). In this study, LMR, ESR, and CRP (although not statistically significant, CRP levels also appear to be higher) differed between the two groups, indicating that these patients had enhanced systemic inflammatory reactions compared to patients with NMOSD without CTD. The view that patients with NMOSD have stronger oxidative stress is based on previous studies showing that the serum albumin level of patients with NMOSD is related to the severity of neurological dysfunction and is lower than that in healthy people (45, 46). Therefore, the serum albumin levels of patients with NMOSD with CTD in this study were lower, suggesting that CTD enhanced the oxidative stress of NMOSD.

There are few studies on the difference between NMOSD with and without CTD, and most of them are single-center studies with small sample sizes. All related studies (for example, the studies listed below), including this one, show that only a few features differ between patients with NMOSD with and without CTD. Zhang et al. found that female patients were more susceptible to NMO/NMOSD with CTD, and patients with NMO/NMOSD with CTD tended to have a higher percentage of CSF-restricted oligoclonal bands and fewer non-specific lesions on brain MRI (12). Yang et al. found that the serum IgG levels were higher, the spinal cord lesions were longer, and the frequency of short transverse myelitis was lower in patients with NMOSD with CTD than those without CTD (13). Xie et al. reported that patients with NMOSD with CTD had more severe neurological impairment, higher positivity rate of serum or cerebrospinal AQP4-IgG, and higher seropositivity of several other autoantibodies than those without CTD (14). Since only AQP4-IgG-positive NMOSD patients were included in this study, we also focused on some studies including AQP4-IgG-negative patients. A noteworthy study has found that the prevalence of SS is higher among AQP4-IgG-positive patients than AQP4-IgG-negative patients, and comorbid SS has a higher relapse frequency among AQP4-IgG-positive patients (47). Although the inclusion criteria and foci of these studies were not exactly the same, their conclusions were generally similar.

There are some limitations to our retrospective cohort study that should be considered in further studies. First, this observational investigation was a single-center small-sample study. Second, according to previous studies, some biomarkers that might be related to CTD, such as serum globulin, IgG, anti-SSA, anti-SSB, ANA, anti-Ro52, and RF, were not detected in this study. Finally, due to the strict evaluation and exclusion criteria of this study, a considerable number of patients, especially AQP4-IgG-negative patients with NMOSD, were excluded, which might have led to bias. In general, these findings need to be validated in more comprehensive, larger, multicenter studies.

## Conclusion

When the first-attack NMOSD patients are complicated with CTD, they have a higher recurrence rate, more recurrences, earlier first recurrence, higher serum Hcy levels, and enhanced systemic inflammatory reactions. Furthermore, Hcy levels may help to screen for CTD in patients with first-attack NMOSD.

## Data availability statement

The original contributions presented in the study are included in the article/Supplementary material, further inquiries can be directed to the corresponding author.

## Ethics statement

The studies involving human participants were reviewed and approved by the Ethics Committee of the First Affiliated Hospital of Zhengzhou University. The patients/participants provided their written informed consent to participate in this study.

## Author contributions

YY: methodology, formal analysis, data curation, writing—original draft, and writing-review and editing. XY and HX: investigation and writing—review and editing. YZ: methodology, investigation, and writing-review and editing. RD and YL: formal analysis. LJ and JT: data curation. WG: CTD diagnose. YJ: conceptualization, methodology, supervision, and funding acquisition. All authors commented on previous versions of the manuscript, read, and approved the final manuscript.

## Conflict of interest

The authors declare that the research was conducted in the absence of any commercial or financial relationships that could be construed as a potential conflict of interest.

## Publisher's note

All claims expressed in this article are solely those of the authors and do not necessarily represent those of their affiliated organizations, or those of the publisher, the editors and the reviewers. Any product that may be evaluated in this article, or claim that may be made by its manufacturer, is not guaranteed or endorsed by the publisher.

## Abbreviations

AQP4: Aquaporin-4
NMO: neuromyelitis optica
NMOSD: neuromyelitis optica spectrum disorder
CTD: connective tissue disease
SLE: systemic lupus erythematosus
SS: Sjogren's syndrome
MRI: magnetic resonance imaging
BD: Behcet's disease
EDSS: Expanded Disability Status Scale
Hcy: Homocysteine
CSF: Cerebrospinal fluid
AQP4-IgG: Aquaporin-4 antibody
ANA: antinuclear antibody
ESR: erythrocyte sedimentation rate
ROC: Receiver operating characteristic
LMR: lymphocyte-to-monocyte ratio
CI: Confidence interval
OR: Odds ratio
SD: Standard deviation
anti-SSA: anti-SSA/Ro antibodies
anti-SSB: anti-SSB/La antibodies
RF: rheumatoid factor.
