# Supplementary material for: Comparative analysis of clinical and imaging data of first-attack neuromyelitis optica spectrum disorders with and without connective tissue disease
Source: Front Neurol. 2022 Aug 25;13:969762. doi: 10.3389/fneur.2022.969762 (PMC9453243; doi:10.3389/fneur.2022.969762)
Supplement: Supplementary file 2 [file Data_Sheet_1.doc]

**CBA of AQP4-IgG measure**

**Detection methods:**

AQP4 anti-antigen gene was transfected into mammals, and the corresponding antigens were specifically expressed in mammalian cells. The co-expression of green fluorescent protein (GFP) was used as the internal reference of the test. The transfected cells were then fixed on a 96-well plate to make antigen tablets, and the specific antibodies in human serum, plasma, or cerebrospinal fluid samples were quantitatively detected by an indirect cotton-padded fluorescence method.

**Results interpretation:**

Under fluorescence microscope, a green-light channel was used to observe the transfection of cells. If the plasmid was successfully transfected, green fluorescence (GFP channel) could be observed. Next, if it was observed that the successfully transfected cell membrane had obvious red fluorescence (AQP4-IgG channel) in the sample hole, it was an antibody positive sample; if it was observed that the successfully transfected cell membrane had no obvious red fluorescence or the unsuccessfully transfected cells had co-color fluorescence, it was a negative sample. The results can be further confirmed by the overlap of green- and red-light channels.

**Test result:**

First judge the negative or positive results according to the above criteria. If the sample is positive, select 3-5 visual fields under the microscope, compare it with the red fluorescence of the quality control substance, and give the positive titer value by comparing with the red fluorescence intensity of the quality control product.

| CBA IIFT result interpretation (serum) | | |
| --- | --- | --- |
| Antibody titer result | The fluorescence intensity can be observed at 1:10 dilution ratio. | Comparison of fluorescence intensity with positive standard samples with titers of 1: 100 and 1: 320 |
| Negative | Negative |  |
| 1：10 | Extremely weak | The fluorescence intensity is much less than 1: 100 |
| 1：30 | Weak | The fluorescence intensity is less than 1: 100 |
| 1：100 | Strong | The fluorescence intensity is equal to 1: 100 |
| 1：300 | Extremely strong | The fluorescence intensity is equal to 1: 300 |
| 1：1000 | Super strong | The fluorescence intensity is greater than 1: 300 |

| CBA IIFT result interpretation（CSF） | | |
| --- | --- | --- |
| Antibody titer result | The fluorescence intensity can be observed at 1:10 dilution ratio. | Comparison of fluorescence intensity with positive standard samples with titers of 1: 100 and 1: 320 |
| Negative | Negative |  |
| 1：1 | Extremely weak | The fluorescence intensity is much less than 1: 100 |
| 1：10 | weak | The fluorescence intensity is less than 1: 100 |
| 1：30 | Medium | The fluorescence intensity is less than 1: 100 |
| 1：100 | Strong | The fluorescence intensity is equal to 1: 100 |
| 1：300 | Extremely strong | The fluorescence intensity is equal to 1: 300 |
| 1：1000 | Super strong | The fluorescence intensity is greater than 1: 300 |
